# ﻿Soil campodeids (Diplura, Campodeidae) of Eastern Europe, in Romanian and Bulgarian reliefs

**DOI:** 10.3897/zookeys.1224.137935

**Published:** 2025-01-31

**Authors:** Alberto Sendra, Cristina Fiera, Jesús Selfa, Pavel Stoev

**Affiliations:** 1 Departament de Didàctica de les Cièncias Experimentals i Socials, Facultat de Magisteri, Universitat de València, València, Spain; 2 Research Team on Soil Biology and Subterranean Ecosystems, Department of Life Sciences, Faculty of Science, Campus Universitario Crta, Universidad de Alcalá, A-2 Km. 33.6, 28805, Alcalá de Henares, Madrid, Spain; 3 Institute of Biology Bucharest, Romanian Academy, 296 Splaiul Independenţei, 060031, Bucharest, Romania; 4 Laboratori d’Investigació d’Entomologia, Departament de Zoologia, Universitat de València, Burjassot, València, Spain; 5 National Museum of Natural History at the Bulgarian Academy of Sciences, Tsar Osvoboditel Blvd. 1, 1000 Sofia, Bulgaria; 6 Pensoft Publishers, Prof. G. Zlatarski Str. 12, Sofia, Bulgaria

**Keywords:** *Campodea*, *Dicampa*, new records, new species, *Paurocampa*, taxonomy

## Abstract

This study presents data on soil campodeids collected in Romania and Bulgaria in recent years. The collection comprises 12 species of genus *Campodea* Westwood, 1842 in total. A new species, Campodea (Dicampa) transylvanica Sendra, **sp. nov.** is described from Zarand and Făgăraș mountains in Romania. Campodea (Campodea) plusiochaeta Silvestri, 1912 is newly recorded for the Romanian fauna, while Campodea (Paurocampa) ruseki Condé, 1966 represents a new record for Bulgaria. New distributional data are also provided for the remaining ten species.

## ﻿Introduction

The first records of Campodeidae species (*Campodeastaphylinus* Westwood, 1842 and *Campodeafragilis* Meinert, 1865) from Romania were published by Vellay (1900) and later cited by Stach (1929). However, both species are now considered misidentifications (Sendra et al. 2012). Years later, in Bulgaria, Silvestri (1931) described three species of campodeids from cave habitats: Campodea (Dicampa) frenata Silvestri, 1931, *Plusiocampabulgarica* Silvestri, 1931, and *Plusiocampabureschi* Silvestri, 1931. Later, Drěnovski (1937) reported three species from soil habitats in Bulgaria: Campodea (Dicampa) malpighii bulgarica Drěnovski, 1937, *Campodeawitoschensis* Drěnovski, 1937, and *Plusiocampamontana* Drěnovski, 1937. However, Paclt (1957, 1969) considered each a nomen nudum, and the first two were respectively assigned to Campodea (Dicampa) frenata Silvesti, 1931 and Campodea (Paurocampa) suensoni Tuxen, 1930 while the third remained without assignation. During 1950s and 1960s, several contributions significantly improved our understanding of the soil and cave Campodeidae of the Carpathians (Romania) and the mountains in Bulgaria (Ionescu 1951, 1955; Rusek 1965a; Paclt 1969). In the 21^st^ century, the diversity of soil species increased to 18 species in Romania (Sendra et al. 2012) and seven species in Bulgaria (Sendra and Georgiev 2021). In addition to the soil-dwelling Campodeidae fauna, nine cave-adapted species have been described or reported: five species from caves in the southern Carpathians (Ionescu 1955; Condé 1991, 1993, 1996; Sendra et al. 2012) and six species from caves in Bulgaria (Silvestri 1931; Bareth and Condé 2001).

The aim of this study is to enhance the understanding of the family Campodeidae within the basal hexapod class Diplura in the Carpathians and Balkan Mountains in Romania and Bulgaria by providing new records and distributional data, describing a new species, and publishing taxonomic and distributional remarks on certain taxa.

In total, 222 specimens from 44 sampling sites in Romania and one site in Bulgaria were examined, based on a collection provided by C. Fiera collected between 2018 and 2021. Additional material from six localities in Bulgaria was collected by Boyan Petrov, Petar Beron, and Pavel Stoev, zoologists at the National Museum of Natural History, Bulgarian Academy of Sciences.

## ﻿Materials and methods

Most of the Diplura specimens were extracted from samples of leaf litter, soil, and mosses using Berlese funnels. The material has been deposited in the private collection of Alberto Sendra, València, Spain (Coll. AS) and most of specimens were mounted on slides using Marc André II medium. These were observed and identified using a phase-contrast microscope, and measurements were taken with an ocular micrometre.

Photomicrography was performed with a stereo microscope (Leica M165C) with an integrated capture system image (LAS v. 4.13) and software LCS Lite, and a compound microscope with a photographic camera K3 C/M and the software LCS Lite. We used the software Helicon Focus to combine photos of a specimen at different levels of focal planes, which helped achieve a more accurate and complete illustration. Several specimens for SEM photography (Hitachi S-4900) were coated with palladium-gold.

The type and studied material are kept at the following institutions:

**Coll. AS** private collection of Alberto Sendra, València, Spain

**IBB**Institute of Biology Bucharest, Romanian Academy

**NMNHS**National Museum of Natural History at the Bulgarian Academy of Sciences.

## ﻿Results

### ﻿ Campodea (Campodea) magna

Taxon classificationAnimalia

Ionescu, 1955

56331F61-157F-5F91-B978-340C3B267D54

#### Material examined.

**Romania** • 1 ex., Bârgău Mountains: Leșu, Bistrița-Năsăud County, 47.289248°N, 24.756708°E, 749 m a.s.l., beach, rarely fir, litter, 02.11.2021, C. Fiera leg.; • 1 ex., RO, Bârgău Mountains: Lunca Ilvei, near Pepiniera Silhoasa, Bistrița-Năsăud County, 47.346245°N, 25.015375°E, 749 m a.s.l., mixed forest (fir and beach), soil, 04.11.2021, C. Fiera leg.; • 2 ex., Doftana Valley: Șotriile, 45.227694°N, 25.729119°E, 609 m a.s.l., beech forest, soil, 18.11.2016, C. Fiera leg.; • 7 ex., Bârgău Mountains: Tureac, site 1, Bistrița-Năsăud County, 47.257408°N, 24.856696°E, 862 m a.s.l., beech forest, soil, 18.08.2018, C. Fiera leg.; • 1 ex., Suceava County: Zamostea-Lunca forest, 47.870137°N, 26.252775°E, 290 m a.s.l., old oak (120 years old, rarely 180 years old), in association with ash, aspen, maple, hornbeam, litter, 08.08.2019, C. Fiera leg.

#### Habitat and distribution.

Soil-dwelling species found in several localities from the southern Carpathians (Ionescu 1955; Sendra et al. 2012) and recently in Bulgaria (Sendra and Georgiev 2021). The species is also known from the northern Anatolia (Sendra et al. 2010).

### ﻿ Campodea (Campodea) plusiochaeta

Taxon classificationAnimalia

Silvestri, 1912

FAE39FDD-6F73-59CC-BB70-2E11DE25AE53

#### Material examined.

**Romania** • 1 ex., Alba County: Cenade, 46.036341°N, 24.007781°E, 436 m a.s.l., vineyards, soil, 10.09.2020, M. Șandor leg.; • 1 ex., Suceava County: Iacobeni, 47.446179°N, 25.311171°E, 895 m a.s.l., mixed forest (fir, larch, hornbeam), soil and litter, C. Fiera leg.

#### Habitat and distribution.

A soil-dwelling species, living under stones or among the alluvial debris (Condé 1960) which is common under barks or moss. It is also found in dry environments, burrows of mammals or gardens, sometimes reaches high altitude in mountains. It is one of the most widespread species collected at many sites of the Euro-Mediterranean region: British Isles (Condé 1961), southern Jutland and southern Scandinavian peninsulas (Silvestri 1912; Arevad 1957; Olsen 1996), North Africa (Condé 1947a, 1953), throughout the Iberian Peninsula (Sendra and Moreno 2004), throughout continental Europe including west, central, and eastern Europe (Silvestri 1912; Pagés 1951; Rusek 1964; Stach 1964; Wygodzinsky 1941; Paclt 1965), Apennine Peninsula (Silvestri 1912; Ramellini 1995; 2000), Balkan Peninsula (Condé 1984), and Anatolia (Sendra et al. 2010). The easternmost localities are in western Russia (Silvestri 1912; Rusek 1965b) close to the 60° parallel.

#### Remarks.

New record for the Romanian fauna.

### ﻿ Campodea (Campodea) taunica

Taxon classificationAnimalia

Marten, 1939

3EA3BDA9-1890-57E7-8081-0137BF9A72B5

#### Material examined.

**Romania** • 1 ex., Dâmbovița County: Springs Complex of Corbii Ciungi, near Corbii Mari County, 44.524361°N, 25.512138°E, 122 m a.s.l., scrubs, soil, M. Manu leg.

#### Habitat and distribution.

A soil-dwelling species that is distributed throughout Central Europe, including France (Husson 1946; Pagés 1951), Central Germany (Paclt 1961), Swiss Alps (Orelli 1956), and reaching as far as the Romanian Carpathians (Ionescu 1951, 1955; Sendra et al. 2012) and Serbia (Blesić 2000a). Surprisingly, it has not been found yet in the Czech Republic or Slovakia. Outside Central Europe, it has been quoted in the Pontic Mountains and the northern part of Anatolia (Sendra et al. 2010).

### ﻿ Campodea (Campodea) wallacei

Taxon classificationAnimalia

Bagnall, 1918

95FF9B9E-4B87-5FC7-AE61-3A762A2A9FA5

#### Material examined.

**Romania** • 4 ex., Bucegi Massif: Sinaia, Prahova County, 45.333328°N, 25.549175°E, 858 m a.s.l., in the city of Sinaia, park, under *Larix* sp., 23.09.2019, C. Fiera and M.W. Weiner leg.

#### Habitat and distribution.

A soil-dwelling species, which is also found in cave habitats (Condé 1956, 1962; Sendra et al. 2013). It is distributed in England (Bagnall 1918), southern Scandinavian Peninsula (Agrell 1944), Maritime Alps (Bareth and Condé 1985; Ramellini 2000), France (Condé 1947b, 1947c, 1950; Pagés 1951), Germany (Christian 2003) and the Dinaric Mountains (Blesić 1998a, 1998b, 2000a, 2001). The species has been recorded from Romania by Ionescu (1951, 1955) and Sendra et al. (2012).

### ﻿ Campodea (Dicampa) apula

Taxon classificationAnimalia

Silvestri, 1912

33E96E55-8688-56E6-95BF-295AE9EC3A08

#### Material examined.

**Romania** • 1 ex., Făgăraș Mountains: Nucșoara, Argeș County, 45.417893°N, 24.733326°E, 1196 m a.s.l., mixed forest (*Fagussylvatica*, *Betulapendula*, *Alnusviridis*, *Sambucus* sp.), litter, 10.11.2021, C. Fiera and I. Vicol leg.

#### Habitat and distribution.

A soil-dwelling species known from Foggia, Italy (Silvestri 1912), the Carpathian Mountains across Slovakia, Poland, and Romania (Ionescu 1951, 1955; Paclt 1961; Szeptycki 1974), and extending to the western border of the Caucasus.

### ﻿ Campodea (Dicampa) campestris

Taxon classificationAnimalia

Ionescu, 1955

86BA1BD2-DE3E-5C22-A93B-BA09F69B94AD

#### Material examined.

**Romania** • 5 ex., Făgăraș Mountains: Sâmbăta de Sus, Brașov County, 45.681608°N, 24.791662°E, 746 m a.s.l., mixed forest (fir and beach), soil, 11.11.2021, C. Fiera and I. Vicol leg.; 1 ex. Doftana Valley: Voila, 45.166241°N, 25.753028°E, 600 m a.s.l., sessile oak and beech, soil, 14.07.2018, C. Fiera leg.

#### Habitat and distribution.

A soil-dwelling species distributed from the southern Carpathians to the Balkan Mountains (Rusek 1965a; Blesić 1984, 1998a, 2000a; Sendra et al. 2012).

### ﻿ Campodea (Dicampa) frenata

Taxon classificationAnimalia

Silvestri, 1931

4BA18A4D-3F84-5463-BC60-E6E587AAA6CA

Fig. 1a−f

#### Material examined.

**Bulgaria** • 3 ex., Vrachanski Balkan Nature Park, hut Purshevitza, 1400 m a.s.l., forest, 12.07.1993, B. Petrov leg.; • 5 ex. Central Balkan National Park, hut Rai, 1250 m a.s.l., foliage, 08.12.1992, B. Petrov leg.; • 5 ex., Western Rhodopes Mts., village Mostovo, under stones, 14.03.1992, B. Petrov leg.; • 2 ex., Western Rila Mts., near Popovski ezera Lakes, 2350 m a.s.l., 25.07.1993, B. Petrov leg.

#### Taxonomic notes.

**Figure 1.**
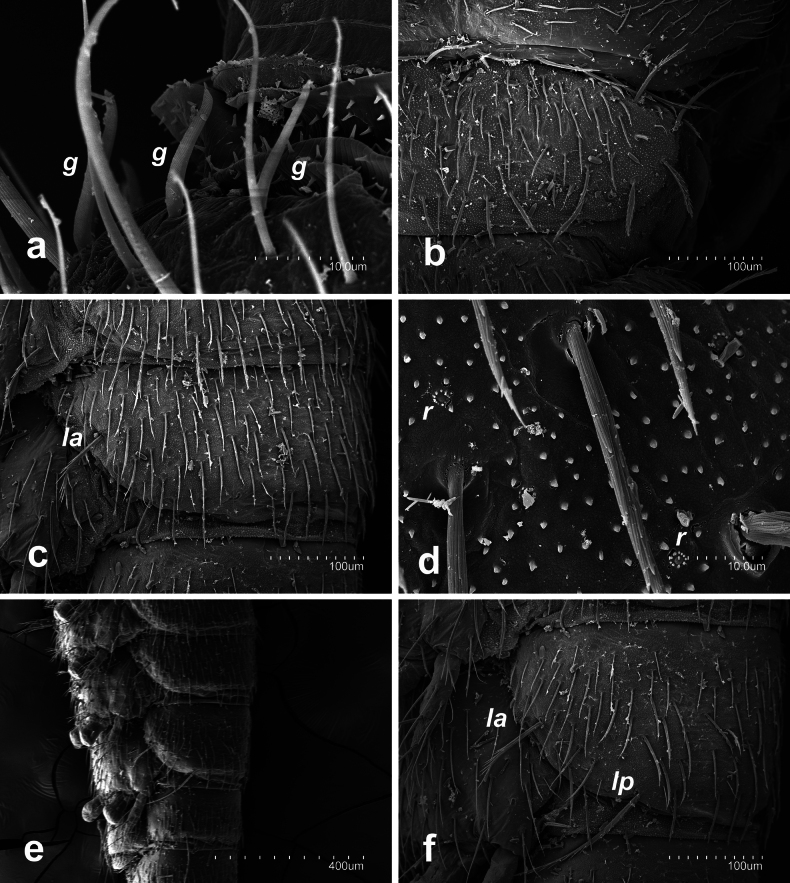
Campodea (Dicampa) frenata Silvestri, 1931, specimen in Coll. AS. **a** lateral anterior view of a medial antennomere **b** pronotum **c** lateral view of abdominal segment 6 **d** detail of pronotum **e** lateral view of abdominal segments 6–8 **f** lateral view abdominal segment 7. Abbreviations: ***g*** gouge sensillum, ***la*** lateral anterior macrosetae, ***r*** rosette gland, *lp* lateral posterior macrosetae.

Specific observations using scanning electron microscopy reveal short and slightly thick gouge sensilla on the antennomeres (Fig. 1a); tergites: dense microdenticles with rosette glands along with well-barbed macrosetae; thick and well-barbed marginal setae, in addition to clothing setae with one or two distal barbs (Fig. 1b−f).

#### Habitat and distribution.

A soil-dwelling species that is only occasionally found in caves (Silvestri 1931; Bareth and Condé 2001). It is distributed throughout the Carpathians Mountains (Ionescu 1955; Rusek 1964), and extends southward into the Balkan Mountains (Paclt 1969; Blesić 1984; 2000a).

### ﻿ Campodea (Dicampa) propinqua

Taxon classificationAnimalia

Silvestri, 1932

8368AC9C-3E4A-5C05-A571-25785899BEBE

Figs 2a–f , 3a−c

#### Material examined.

Romania • 1 ex., Bârgău Mountains: Valea Mare, Bistrița-Năsăud County, 47.481778°N, 24.999045°E, 762 m a.s.l., spruce forest, soil, 04.11.2021, C. Fiera leg.; • 1 ex., Bârgău Mountains: Șanț, Bistrița-Năsăud County, 47.50723°N, 24.970762°E, 826 m a.s.l., cutted spruce forest, litter, 04.11.2021, C. Fiera leg.; • 15 ex., Brașov County: Pârâul Rece, 45.512108°N, 25.507398°E, 1073 m a.s.l., spruce forest, litter, 12.11.2021, C. Fiera and I. Vicol leg.; • 5 ex., Făgăraș Mountains: Cârtișoara, Sibiu county, 45.670567°N, 24.590975°E, 927 m a.s.l., beech forest, soil, 10.10.2020, C. Fiera and I. Vicol leg.; • 5 ex., Făgăraș Mountains: Turnu Roșu, Sibiu county, 45.616342°N, 24.323312°E, 704 m a.s.l., mixed forest (spruce, fir, beech), predominantly spruce. litter, 11.11.2021, C. Fiera and I. Vicol leg.; • 1 ex., Făgăraș Mountains: Cârtișoara, Sibiu county, 45.671965°N, 24.791662°E, 808 m a.s.l., beach forest, litter, 11.11.2021, C. Fiera and I. Vicol leg.; • 1 ex., Făgăraș Mountains: near Berivoi Monastery, Brașov County, 45.687445°N, 24.970958°E, 709 m a.s.l., beech forest, rarely fir, litter, 12.11.2021, C. Fiera and I. Vicol leg.; • 3 ex., Suceava County: Iacobeni, 47.446179°N, 25.311171°E, 895 m a.s.l., mixed forest (fir, larch, hornbeam), soil and litter, C. Fiera leg.; • 2 ex., Gorj County: Rânca locality, 45.301415°N, 23.680959°E, 1622 m a.s.l., spruce forest, soil and litter, 17.06.2021, C. Fiera leg.; • 1 ex., Zarand Mountains, site 1, Căsoaia, near Arăneag, Arad county, 46.225324°N, 21.764489°E, 226 m a.s.l., mixed forest (*Abiesalba*, *Fagussylvatica*, *Quercusfrainetto*, *Carpinusbetulus*, *Acercampestre*), soil, 10.11.2020, C. Fiera and I. Vicol leg.; • 1 ex., Zarand Mountains, site 4, Milova, Arad county, 46.124375°N, 21.801121°E, 191 m a.s.l., mixed forest (*Piceaabies*, *Fagussylvatica*, *Pinusnigra*, *Acercampestre*, *Quercus* sp., *Acespseudoplatanus*), soil, 11.11.2020, C. Fiera leg. and I. Vicol; • 2 ex., Zarand Mountains, site 5, Bârzava, Arad county, 46.127396°N, 21.986602°E, 168 m a.s.l., mixed forest (*Fagussylvatica*, *Quercuspetraea*, *Aviumcerasus*), soil, 11.11.2020, C. Fiera and I. Vicol leg.; • 1 ex., Zarand Mountains, site 6, Conop, Arad county, 46.098845°N, 21.903658°E, 165 m a.s.l., mixed forest (*Quercuscerris*, *Q.frainetto*, *Acercampestre*, *Fagussylvatica*, *Ligustrumvulgare*, *Robiniapseudocacia*, *Sorbus* sp., *Carpinusbetulus*, *Sorbusterminalis*), soil, 11.11.2020, C. Fiera and I. Vicol leg. **Bulgaria** • 1 ♂, 1 ♀, Pirin Mts., 6 km of Predela, MSS trap, alt. 676 m a.s.l., 16.06.2006, P. Stoev, B. Petrov leg.

#### Taxonomic notes.

The morphological taxonomic features observed in the studied specimens under an optical microscope show no differences from the Iberian specimens. However, a molecular analysis should be conducted to confirm whether these geographically distant populations belong to the same species.

**Figure 2.**
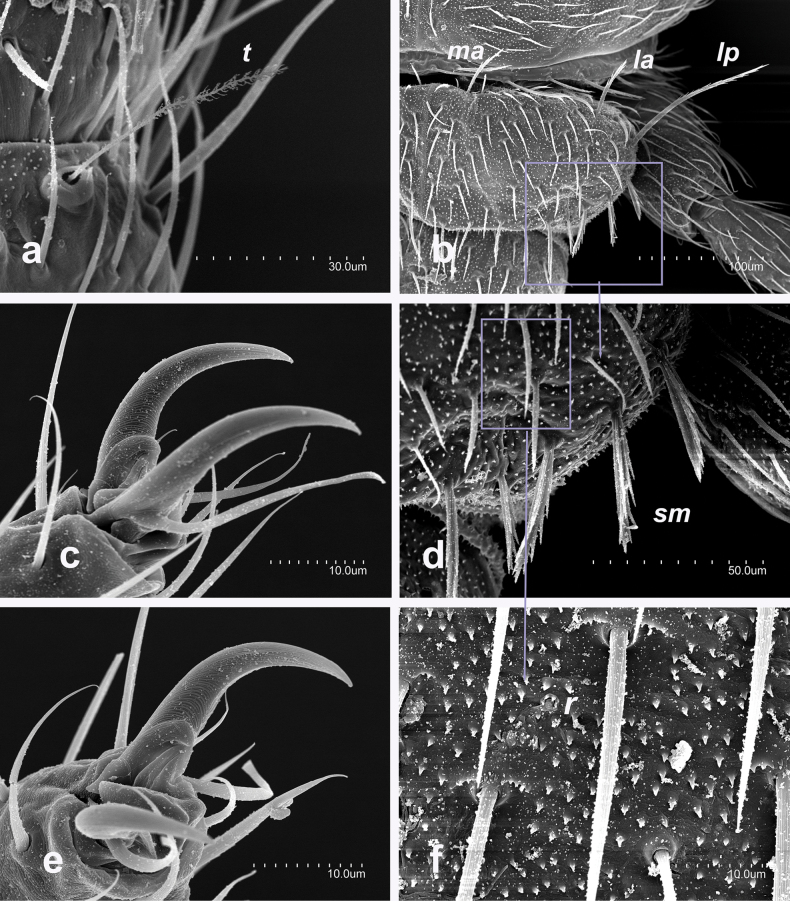
Campodea (Dicampa) propinqua Silvestri, 1932, specimen in Coll. AS**a** latero-anterior view of third antennomere **b** pronotum **c** pretarsus metathoracic leg **d** latero-anterior view of pronotum **e** pretarsus metathoracic leg **f** detail pronotum. Abbreviations: ***t*** trichobothria, ***ma*** medial anterior macrosetae, ***la*** lateral anterior macrosetae, ***lp*** lateral posterior macrosetae, ***sm*** marginal setae, ***r*** rosette gland.

**Figure 3.**
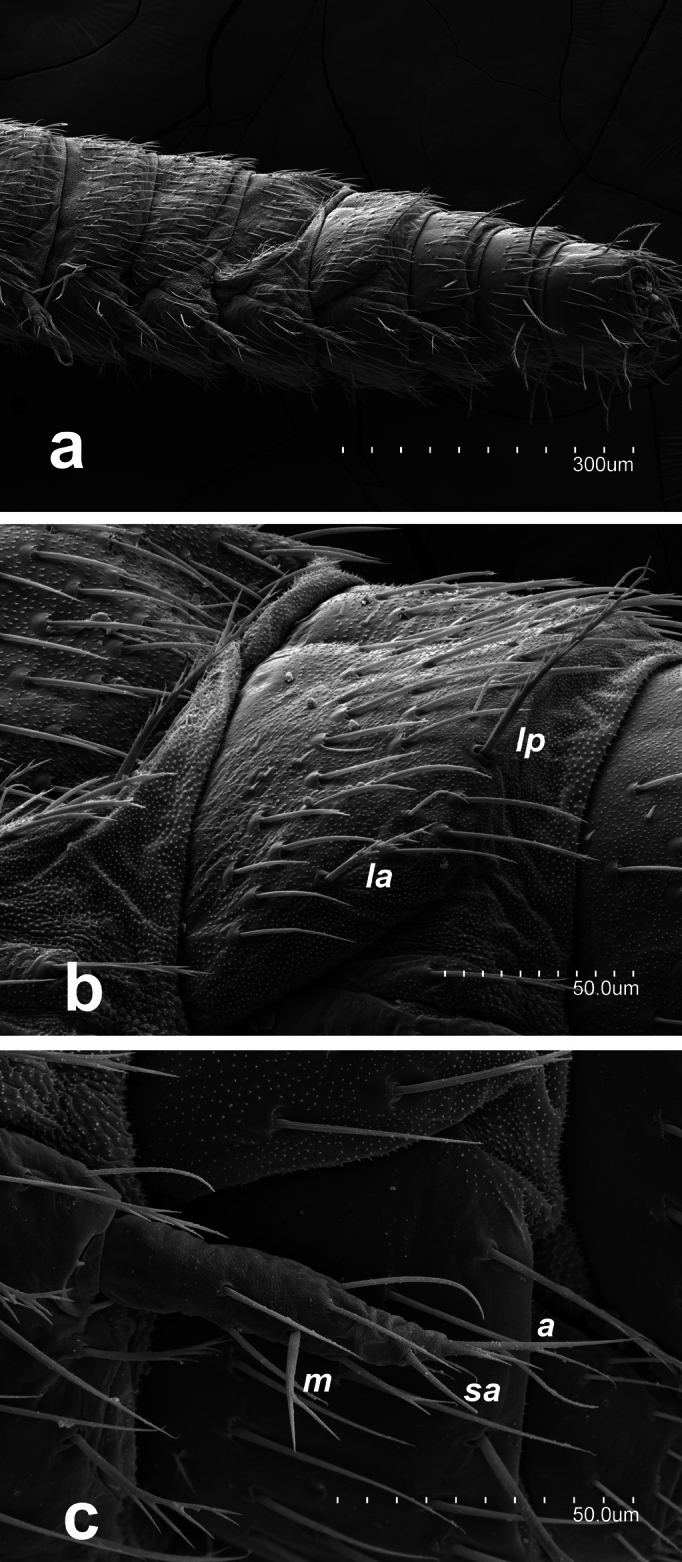
Campodea (Dicampa) propinqua Silvestri, 1932, specimen in Coll. AS**a** abdomen segments 4–10 **b** lateral view abdominal segment 7 **c** stylus abdominal segment 7. Abbreviations: ***la*** lateral anterior macrosetae, ***lp*** lateral posterior macrosetae, ***m*** medial setae, ***sa*** subapical setae, ***a*** apical setae.

Specific observations using scanning electron microscopy reveal large embase of antennal trichobothria (Fig. 2a), dense microdenticles (Fig. 2b, d) including rosette-type glands on all tergites (Fig. 2f), well-barbed macrosetae (Fig. 2b), and clothing setae with a single distal barb (Fig. 3a, b). Additionally, the stylus setae are smooth, with the apical one featuring two long basal denticles (Fig. 3c). Claws are simple, with a protuberance between them (Fig. 2c, e).

#### Habitat and distribution.

A soil-dwelling species known from a single locality in the western Subbaetic Mountains, southern Iberian Peninsula (Silvestri 1932a), and inhabiting colluvial scree slopes in central Iberia (Sendra et al. 2017). Reported from Romania by Ionescu (1951, 1955) and Italy by Ramellini (1990).

### ﻿ Campodea (Dicampa) sprovierisprovieri

Taxon classificationAnimalia

Silvestri, 1932

BB4F5DE2-F494-5721-A159-A3B4F9A9F9DF

#### Material examined.

**Romania**• 1 ♀, Bârgău Mountains: Leșu, Bistrița-Năsăud County, 47.289248°N, 24.756708°E, 749 m, beach, rarely fir, litter, 02.11.2021, C. Fiera leg.; • 1 ♀, 1 ♂, Brașov County: Pârâul Rece, 45.515038°N, 25.503134°E, 994 m a.s.l., spruce forest, soil, 14.07.2018, C. Fiera leg.; • 2 ♂, 3 ♀, 1 juv., Vrancea County: Brădăcești (near Nereju), 45.697641°N, 26.671177°E, 623 m a.s.l., coniferous forest (*Pinus* sp.), soil, 27.05.2021, C. Fiera leg.; • 1 ♂, 2 ♀, Suceava County: Iacobeni, 47.446179°N, 25.311171°E, 895 m a.s.l., mixed forest (fir, larch, hornbeam), soil and litter, C. Fiera leg.; • 1 ♀, Suceava County: Pătrăuți forest near Răuțeni locality, 47.840511°N, 25.056427°E, 344 m a.s.l., mixed forest (beech, oak, and hornbeam), soil, 13.04.2021, C. Fiera leg.; • 1 juv. Brașov County: Prejmer, site 1, 45.730329°N, 25.737091°E, 510 m a.s.l., mixed forest (maple, lime, hornbeam), soil, 15.11.2018, C. Fiera and Weiner M.W. leg.; • 3 ♀, 2 ♂, 3 juvs, Brașov County: Prejmer, site 3, 45.75037°N, 25.723362°E, 510 m a.s.l., oak forest (*Quercuscerris*), 100 years old, 15.11.2018, C. Fiera and Weiner M.W. leg.; • one juv., Brașov County: Prejmer, site 4, 45.753624°N, 25.707257°E, 501 m a.s.l., oak forest (*Quercuscerris*), less than 70 years old, 15.11.2018, C. Fiera and Weiner M.W. leg.; • 4 ex., Cheile Zugreni, between Bistriţei and Giumalău mountains, 47.407101°N, 25.545952°E, 770 m a.s.l., pine and spruce forest, 18.08.2018, C. Fiera leg.; • 1 ♂, Suceava County: Dornișoara, 47.213982°N, 25.06118°E, 1109 m a.s.l., spruce forest, 18.08.2018, C. Fiera leg.; • 1 ♀, Brașov County, Hărman, site 2, 45.734179°N, 25.671031°E, 508 m a.s.l., peatbog with oak, soil, 16.11.2018, C. Fiera and Weiner M.W. leg.; • 5 ♂, 6 ♀, 7 juvs and 3 specimens kept for DNA, Bucegi Massif: Sinaia, Prahova county, site 1, 45.351264°N, 25.521893°E, 1288 m a.s.l., spruce forest, soil, 14.07.2018, C. Fiera leg.; • 5 ♀, 5 ♂, 7 juvs and 4 specimens kept for DNA, Bucegi Massif: Sinaia, Prahova county, site 2, 45.357541°N, 25.516975°E, 1386 m a.s.l., spruce forest, soil, 14.07.2018, C. Fiera leg.; • 3 juvs, Buzău County: Siriu, 45.559882°N, 26.178259°E, 658 m a.s.l., beech forest, soil, 27.06.2020, C. Fiera leg.; • 2 ♀, 2 juvs, Doftana Valley: Șotriile, 45.227694°N, 25.729119°E, 609 m a.s.l., beech forest, soil, 18.11.2016, C. Fiera leg.; • 2 ♂, 1 ♀, one juv. and one specimen kept for DNA, Bârgău Mountains: Tureac, site 1, Bistrița-Năsăud County, 47.257408°N, 24.856696°E, 862 m a.s.l., beech forest, soil, 18.08.2018, C. Fiera leg. **Bulgaria** • 1 ♀, 4 juvs, Pirin Mountains, Bansko, 41.77552°N, 23.439216°E, 1784 m a.s.l., spruce forest, soil, 13.08.2018, C. Fiera leg.

#### Habitat and distribution.

A soil-dwelling species which is widely spread around the Balkan Peninsula (Ionescu 1955; Rusek 1965b; Condé 1984; Sendra et al. 2012) and Anatolia (Sendra et al. 2006, 2010), including several Aegean islands (Silvestri 1933; Condé 1984).

### ﻿ Campodea (Dicampa) transylvanica

Taxon classificationAnimalia

Sendra sp. nov.

24B80BD5-31B0-5C62-BB65-31A0EE73A113

https://zoobank.org/7655C15C-D6CB-4D70-AD16-DA30DA48A7FC

**Figure 4.**
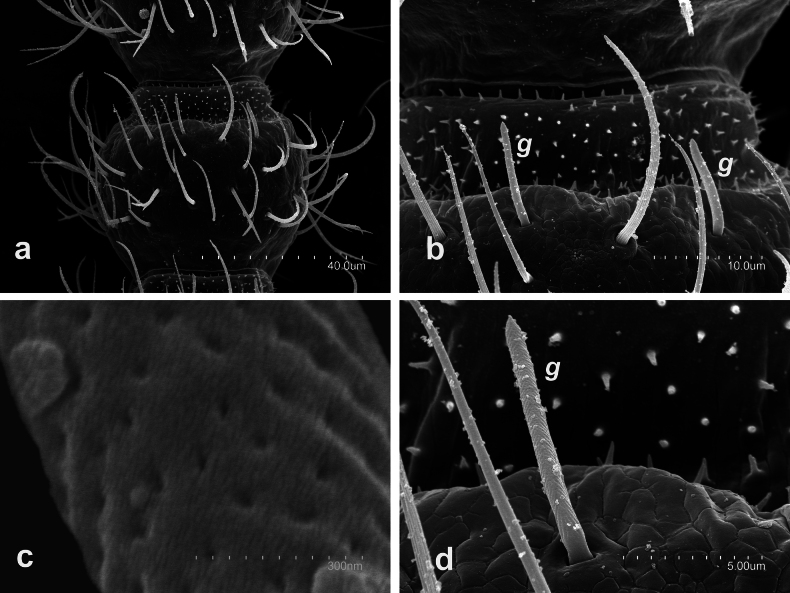
Campodea (Dicampa) transylvanica Sendra, sp. nov., specimen in Coll. AS**a** medial antennomere **b** latero-anterior view of a medial antennomere **c** detail of gouge sensillum **d** gouge sensillum on antennomere. Abbreviation: ***g*** gouge sensillum.

**Figure 5.**
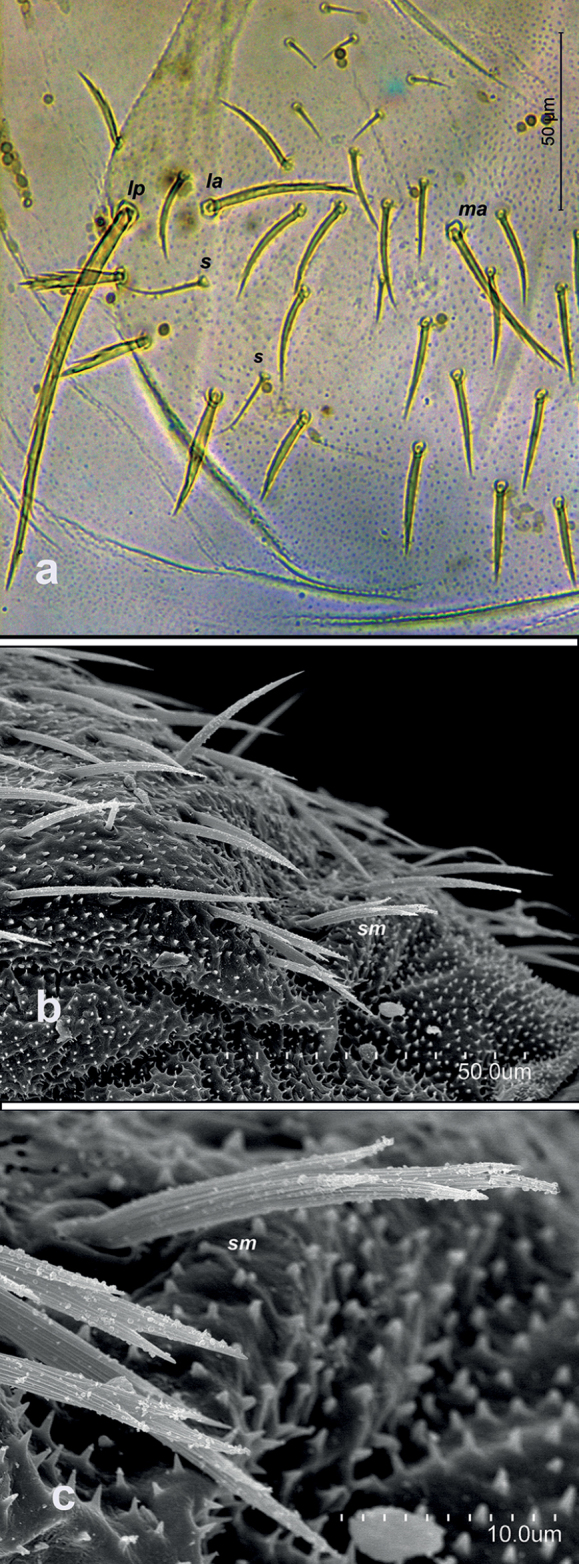
Campodea (Dicampa) transylvanica Sendra, sp. nov., specimens in Coll. AS.) **a** pronotum **b** latero-posterior view of pronotum **c** detail of latero-posterior pronotum. Abbreviations: ***ma*** medial anterior macrosetae, ***la*** latero-anterior macrosetae, ***lp*** latero-posterior macrosetae, ***s*** sensillum, ***sm*** marginal setae.

**Figure 6.**
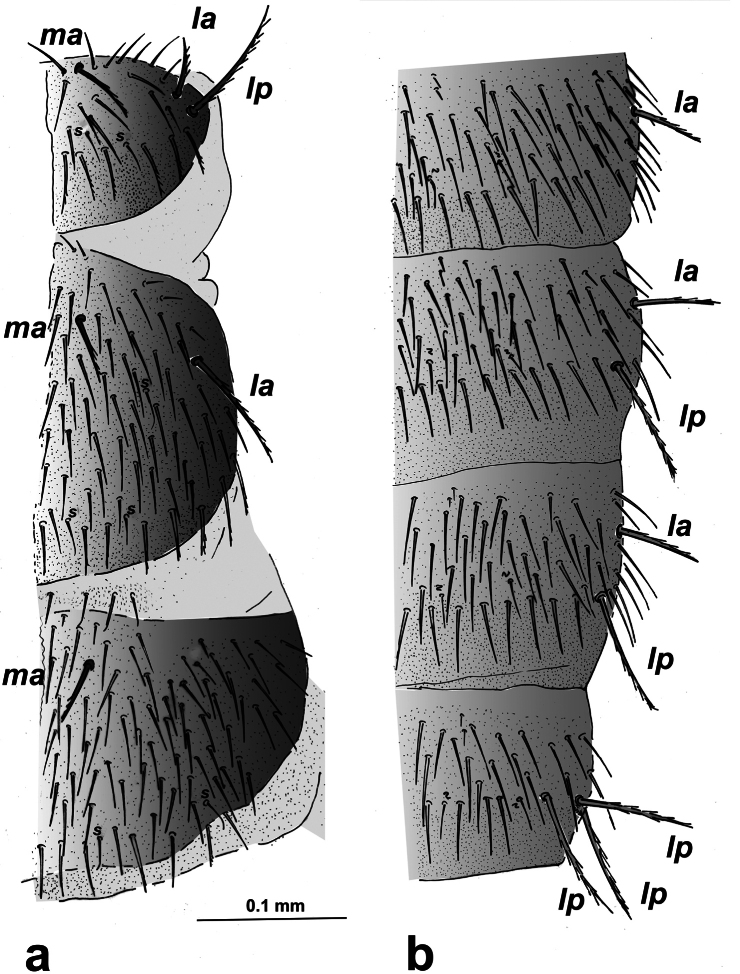
Campodea (Dicampa) transylvanica Sendra, sp. nov. Holotype IBB-CTR1 **a** pronotum, mesonotum, and metanotum **b** urotergites V–VII. Abbreviations: ***ma*** medial anterior macrosetae, ***la*** latero anterior macrosetae, ***lp*** latero posterior macrosetae, ***s*** sensillum.

Figs 4a−d , 5a−c , 6a, b

#### Type material.

***Holotype*. Romania** • ♂; Turnu Roșu (Sibiu County), Făgăras Mountains (RO Carpathians), soil, 704 m. elevation; mixed forest (spruce, fir, beech) (predominant spruce); 11 November 2021, Fiera C. and I. Vicol leg.; labelled holotype IBB-CTR1. ***Paratypes*. Romania** • 1 ♀, 2 ♂♂, 2 juvs, Turnu Roșu, Făgăraş Mountains (RO Carpathians), Făgăraş, soil, 704 m elevation, mixed forest (spruce, fir, beech) (predominant spruce), 11 November 2021, Fiera C. and I. Vicol leg., labelled IBB-CTR2-5; • 1 ♀, Turnu Roșu, Făgăraş Mountains (RO Carpathians), litter, 704 m. elevation, mixed forest (spruce, fir, beech) (predominant spruce), 11 November 2021, Fiera C. and I. Vicol leg., labelled IBB-CTR6; • 1 ♂, Cârtișoara, Făgăras Mountains (RO Carpathians), litter, beach, 11 November 2021, Fiera C and Vicol I. leg., labelled IBB-CTR7; • 3 ♀♀, Cârtișoara, Făgăras Mountains (RO Carpathians), soil, beach, 11 November 2021, Fiera C. and I. Vicol leg., labelled NMNHS-10832-10834; • 1 juv., Radna (Arad County), Zarand Mountains (RO Carpathians), *Quercuscerris*, *Q.frainetto*, *Betulapendula*, 12 November 2020, Fiera C. and I. Vicol leg., labelled IBB-CTR8; • 1 ♀, 1 ♂, Conop (Arad County), Zarand Mountains (RO Carpathians), *Quercuscerris*, *Q.frainetto*, *Acercampestre*, *Fagussylvatica*, *Ligustrumvulgare*, *Robiniapseudoacacia*, *Sorbus* sp., *Carpinusbetulus*, *Sorbusterminalis*, 11 November 2021, Fiera C. and I. Vicol leg., labelled IBB-CTR9-10.

#### Other material.

**Romania** • 1 ♀, Turnu Roșu, Făgăraş Mountains (RO Carpathians), Făgăraş, soil, 704 m elevation, mixed forest (spruce, fir, beech) (predominant spruce), 11 November 2021, Fiera C. and I. Vicol leg, Coll AS.

#### Description.

***Body***. Length 1.9–2.7 mm in male; 2.3–3 mm in females; 1.4–1.7 mm in juveniles. Epicuticle with microdenticles under optical microscope (Fig. 5a) and dense microdenticles under scanning electron microscope (Fig. 5b, c); body with smooth short clothing setae (Fig. 5a−c).

***Head*.** Antennae with 15–19 antennomeres in juveniles and adults, 0.53–0.47 shorter than length of the body in juveniles and 0.45–0.31 in adults; central antennomeres as long as wide with one proximal whorl of bifurcated macrosetae and one distal whorl of smooth macrosetae and uneven short smooth setae; in addition to a single distal whorl of ≤ 4–6 gouge sensilla of 5–6 µm long (Fig. 4a−d). Proximal antennomeres with typical trichobothria disposition and with small bacilliform sensillum on third antennomere in dorsal position, between *b–c* macrosetae. Plain frontal process with one anterior macrosetae, longer than clothing setae. Three macrosetae each with two or three barbs along each side of insertion line of antennomere with length ratios of *a*/*i*/*p*, 11/13/14, respectively, in holotype. Large suboval labial palps, each with small latero-external sensillum near two gard setae and five normal setae on anterior portion, ≤ 60 neuroglandular setae in medial and posterior positions.

***Thorax*.** Thoracic macrosetae distribution: pronotum has 1+1 *ma*, 1+1 *la*, 1+1 *lp* macrosetae; mesonotum has 1+1 *ma*, 1+1 *la* macrosetae (Figs 5a, 6a). All macrosetae longer than other setae with barbs in distal ½–3/4 marginal setae barbed and longer than clothing setae. Short legs, metathoracic legs reach border of fourth abdominal segment. Calcars with two or three long barbs in middle. Each tarsus with two separated ventral rows of slightly and thicker smooth setae among clothing setae. Three long smooth dorsal tarsal and one ventral setae. Subequal simple claws and with smooth lateral processes.

***Abdomen*.** Distribution of abdominal macrosetae on tergites (Fig. 6b) shows 1+1 la on V, 1+1 *la*, 1+1 *lp* on VI–VII; 3+3 *lp* on VIII and 5+5 *lp* on IX abdominal; *la* macrosetae with barbs in distal ½–1/3 and *lp* macrosetae bear barbs along distal 4/5. Urosternite I with 7+7 macrosetae; urosternites II to VII with 4+4 macrosetae; urosternite VIII with 1+1 macrosetae; urosternal macrosetae of bifurcated, tri or quadrifurcated. Stylus setae with smooth subapical setae, bifurcated ventromedial seta and with two long basal barbs on apical seta.

Secondary sex features. Female urosternite I with subcylindrical appendages, each bearing ≤ 12 glandular *a*1 setae in apical field. Male urosternite I with subtrapezoidal appendages, each with apical field of ≤ 17 glandular *a*1 setae; a continuous posterior field of ≤ about 100 *g1* glandular setae arranged in 1–3 rows. Two incomplete cerci with basal article plus five primary articles. Internal smooth macrosetae or with one distal barb in proximal articles and other macrosetae with four or five distal barbs; primary articles with up to three whorls of barbed macrosetae, and uneven short smooth setae.

#### Taxonomic affinities.

The distribution of urotergal macrosetae in Campodea (Dicampa) transylvanica Sendra, sp. nov. from the Romanian Carpathians matches that of Campodea (Dicampa) plagiaria Silvestri, 1932 from the Baetic and Riff mountains (Silvestri 1932b). However, Campodea (D.) transylvanica Sendra, sp. nov. differs from C. (D.) plagiaria in several taxonomic features: 15–19 antennomeres in Campodea (D.) transylvanica Sendra, sp. nov. instead of 26–28 antennomeres in soil populations of Campodea (D.) plagiaria; sensillum on the third antennomere in tergal position in C. (D.) transylvanica Sendra, sp. nov. instead of ventral in C. (D.) plagiaria; apical barbs on marginal notal setae in C. (D.) transylvanica Sendra, sp. nov. instead of thin pine marginal setae in C. (D.) plagiaria.

#### Etymology.

Named after Transylvania, a historical and cultural region in Central Europe that encompasses central Romania.

#### Habitat and distribution.

A soil-dwelling species that is found in mixed forests in Zarand and Făgăraș mountains. In two localities it co-occurs with Campodea (Dicampa) propinqua Silvestri, 1932.

### ﻿ Campodea (Paurocampa) ruseki

Taxon classificationAnimalia

Condé, 1966

EDA89E37-D797-55B1-BCC8-58B01118AA8E

Fig. 7a, b

#### Material examined.

**Bulgaria** • 2 ♂♂, 1 ♀, Pirin Mountains, Hut Kamenitsa, 1800 m a.s.l., 15.06.1988, P. Beron leg.

#### Taxonomic notes.

**Figure 7.**
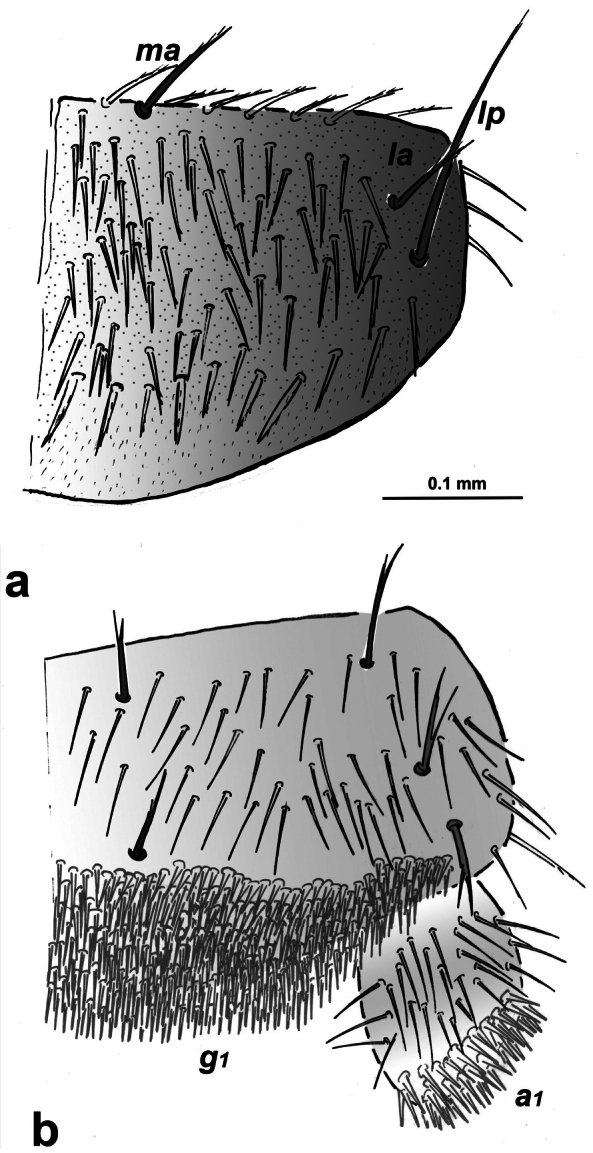
Campodea (Paurocampa) ruseki Condé, 1966 **a** pronotum **b** first urosternite of a male. Abbreviations: ***ma*** medial anterior macrosetae, ***la*** latero-anterior macrosetae, ***lp*** latero-posterior macrosetae, ***g_1_*** glandular setae type g1, ***a_1_*** glandular setae type a1.

Observations under the microscope of the studied material have shown several previously unknown features not mentioned in its original description (Condé 1956, 1966). The antennae have 27 antennomeres in a 3.8 mm female, and 25 in 3.95 mm and 4.2 mm males. The apical antennomere has four simple spheroidal olfactory chemoreceptors on the cupuliform organ. A large bacilliform sensillum is present on the third antennomere in tergal position (between b-c macrosetae). Notal tergites bear microdenticles, and the clothing setae are either smooth or have a distal tiny barb (Fig. 7a). The marginal setae are slightly longer and thicker than clothing setae, with a few bars on distal half to two-thirds. The pronotal macrosetae have a few thin tiny barbs on the distal half, with the longest *lp* macrosetae with one or two thin tiny barbs at the distal position. No trochanteral setae were observed in any of the specimens studied.

The male urosternite I (Fig. 7b) features slightly spherical appendages, each with an apical field containing ≤ 40 glandular *a*1 setae; a continuous posterior field of ~≤ 240 *g1* glandular setae arranged in 7–9 rows.

#### Remarks.

New record for Bulgaria.

#### Habitat and distribution.

A soil-dwelling species that inhabits high altitudes. It is known from two localities in the Austrian Alps (Condé 1954, 1966) and a single locality in the Pirin Mountains of Bulgaria.

### ﻿ Campodea (Paurocampa) suensoni

Taxon classificationAnimalia

Tuxen, 1930

93382CDC-CD7B-512A-95D6-3701D91B3319

Fig. 8a–f

#### Material examined.

**Romania** • 3 ♂, 1 ♀, 4 juvs, Bârgău Mountains: Leșu, Bistrița-Năsăud County, 47.289248°N, 24.756708°E, 749 m, beach, rarely fir, litter, 02.11.2021, C. Fiera leg.; • 2 juvs, Bârgău Mountains: near Ilva Mică, Bistrița-Năsăud County, 47.328709°N, 24.702183°E, 494 m a.s.l., beach forest, litter, 02.11.2021, C. Fiera leg.; • 1 ♀, Bârgău Mountains: Tureac, site 8, Bistrița-Năsăud County, 47.257365°N, 24.857551°E, 872 m a.s.l., beach forest, soil, 03.11.2021, C. Fiera leg.; • 2 ♂, 2 ♀, Făgăraș Mountains: Nucșoara, Argeș County, 45.417893°N, 24.733326°E, 1196 m a.s.l., mixed forest (*Fagussylvatica*, *Betulapendula*, *Alnusviridis*, *Sambucus* sp.), litter, 10.11.2021, C. Fiera and I. Vicol leg.; • 1 ♂, 1 ♀, 2 juvs, Suceava County: Iacobeni, 47.446179°N, 25.311171°E, 895 m a.s.l., mixed forest (fir, larch, hornbeam), soil and litter, C. Fiera leg.; • 1 ♀, Argeș County: near Râușor Lake, 45.397983°N, 25.056427°E, 431 m a.s.l., rocks, soil, 26.07.2020, C. Fiera leg.; • 3 ♀, 6 ♂, 20 juvs and one specimen kept for DNA, Bârgău Mountains: Tureac, site 1, Bistrița-Năsăud County, 47.257408°N, 24.856696°E, 862 m a.s.l., beech forest, soil, 18.08.2018, C. Fiera leg.; • one juv., Bistriţa-Năsăud County: Valea Străjii near Tiha Bârgăului, 47.211925°N, 24.879244°E, 701 m a.s.l., beech forest, soil, 18.08.2018, C. Fiera leg.; • 2 ♀, Zarand Mountains, site 1, Căsoaia, near Arăneag, Arad county, 46.225324°N, 21.764489°E, 226 m a.s.l., mixed forest (*Abiesalba*, *Fagussylvatica*, *Quercusfrainetto*, *Carpinusbetulus*, *Acercampestre*), soil, 10.11.2020, C. Fiera and I. Vicol leg. Bulgaria • 2 ♂♂, 2 ♀♀, 3 juvs, Pirin Mountains, above Bansko, 41.77552°N, 23.439216°E, elevation 1784 m, spruce forest, soil, 13.08.2018, leg. C. Fiera; • 2 ex., Pirin Mt., Popina laka Lake, 18 km from Sandanski 1200–1400 m a.s.l., 15.06.1988. P. Beron leg.

#### Taxonomic notes.

**Figure 8.**
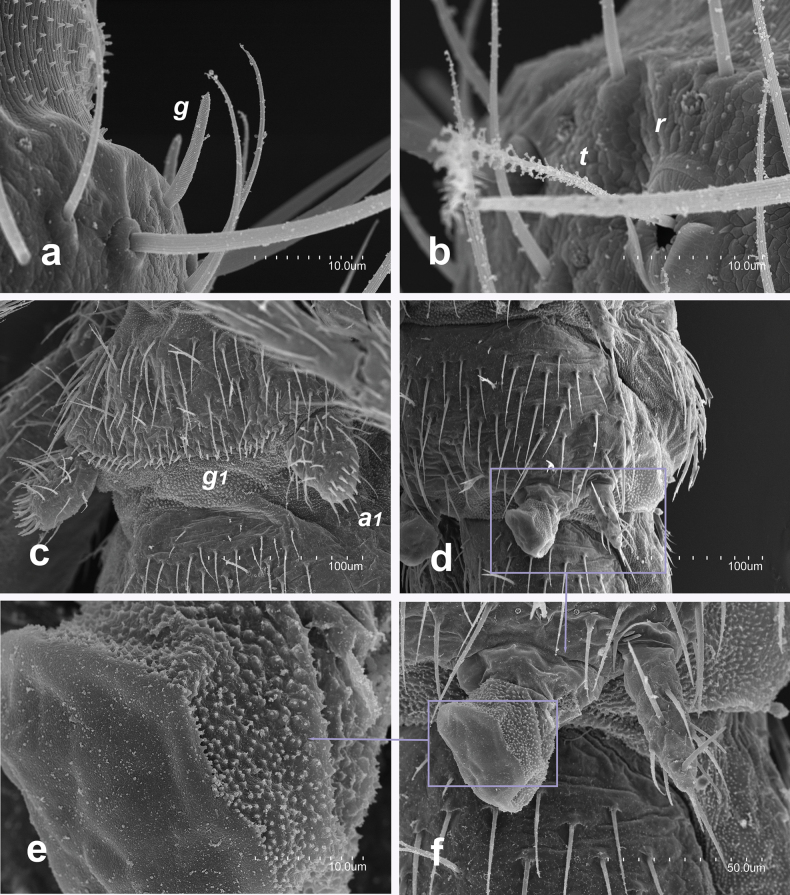
Campodea (Paurocampa) suensoni Tuxen, 1930 **a** antero-lateral view of a medial antennomere **b** distal portion of third antennomere **c** first urosternite of a male **d** sixth urosternite **e** detail exerted vesicle **f** latero-posterior view of sixth urosternite. Abbreviations: ***g*** gouge sensillum, ***t*** trichobothria, ***r*** rosette gland, ***g_1_*** glandular setae type g1, ***a_1_*** glandular setae type a1.

Observations under electronic scanning microscope shown several taxonomic morphological characters in details: short gouge sensilla on antennomeres (Fig. 8a); trichobothria with large embase (Fig. 8b); male urosternite I (Fig. 8c) with subspherical appendages, each with an apical field containing glandular *a_1_* setae in addition to a continuous posterior field of *g_1_* glandular setae arranged in several rows; and, urosternites I–VII with short stylus and large exerted vesicles with two differentiated cuticle areas (Fig. 8d−f).

#### Habitat and distribution.

A soil-dwelling species that is found also at the entrances of caves and in their deeper zones when abundant organic matter is available (Condé 1974; Sendra et al. 2012). It is common and well-distributed in Central and Eastern Europe (Tuxen 1930; Condé, 1954, 1966; Paclt and Rusek 1961; Condé 1956; Rusek 1965b; Paclt 1961, 1969; Blesić 1984, 1997, 2000b, 2001; Christian 1992; Sendra et al. 2012), extending its distribution southward into central Italy (Condé 1966; Ramellini 2000).

## ﻿Discussion

Campodeids and other Diplura families have been poorly sampled worldwide, despite their omnipresence in soils and subterranean spaces, including caves accessible or not by humans, as noted by Racovitza (1907) and Sendra (2023). Fortunately, thanks to the Emil Racovita Institute of Speleology in Romania and the National Museum of Natural History in Sofia, the soil and caves campodeid fauna of these two countries has been relatively well documented, primarily in five major contributions (Silvestri 1931; Ionescu 1955; Bareth and Condé 2001; Condé 1996; Sendra et al. 2012). Our study has increased the total number of soil and cave campodeids for Bulgaria and Romania from 19 to 22 species, which include three novelties: Campodea (Paurocampa) ruseki (new record for Bulgaria), *Campodeaplusiochaeta* (new record for Romania), and one new species, Campodea (Dicampa) transylvanica Sendra, sp. nov. (Table 1). The soil campodeids in the studied region belong to four genera: *Campodea* Westwood, 1842 with three subgenera *Campodea* s. str. Westwood, 1842 (eight species), *Dicampa* Silvestri, 1932 (nine species) and *Paurocampa* Silvestri, 1932 (two species), *Eutrichocampa* Silvestri, 1902 (one species), *Litocampa* Silvestri, 1933 (one species), and *Plusiocampa* Silvestri, 1912 (one species). This biodiversity is higher in terms of both species and genera, including subgenera, than that of other European regions at the same latitude such as Germany, which has a land area similar to that of Bulgaria and Romania combined, and a total of 13 *Campodea* s. str. species. However, France, with nearly twice the land area of Romania and Bulgaria together, has 47 soil and cave species, primarily from the subgenus Campodea (32 species) as well as the subgenera *Dicampa* (four species), *Monocampa* Silvestri, 1932 (four species), and *Paurocampa* (two species) plus the genera *Eutrichocampa* (one species), *Litocampa* (two species), *Plusiocampa* (one species), and *Podocampa* Silvestri, 1932 (one species) (Sendra and Reboleira 2020; Sendra et al. 2020). Furthermore, nearby countries such as continental Greece, one third of the combined area of Bulgaria and Romania has eleven species: Campodea (five species), one species in subgenus *Dicampa*, one in the subgenus Paurocampa, one in *Helladocampa* Condé, 1984, plus three in *Plusiocampa*. In Serbia and Macedonia, with a similar combined area size as continental Greece, there are 19 species distributed within four genera: *Campodea* (8 *Campodea* s. str. species, 5 *Dicampa* species, and 2 *Paurocampa* species), *Eutrichocampa* with two species, *Podocampa* with two species, and finally *Cestocampa* with one species (Sendra and Reboleira 2020; Sendra et al. 2020).

**Table 1.** Soil campodeid species from Bulgaria and Romania.

Soil species	Country	Reference
Campodea (Campodea) fragilis Meinert, 1865	Romania	Ionescu 1955
Campodea (Campodea) magna Ionescu, 1955	Bulgaria, Romania	Ionescu 1955; Sendra et al. 2012; Sendra and Georgiev 2021
Campodea (Campodea) plusiochaeta Silvestri, 1912	Romania	This study - new species for Romania
Campodea (Campodea) pseudofragilis Condé, 1984	Romania	Ionescu 1951, 1955; Sendra et al. 2012
Campodea (Campodea) taunica Marten, 1930	Romania	Ionescu 1951, 1955; Sendra et al. 2012
Campodea (Campodea) tuxeni Wygodzinsky, 1941	Romania	Sendra et al. 2012
Campodea (Campodea) vihorlatensis Paclt, 1961	Romania	Sendra et al. 2012
Campodea (Campodea) wallacei Bagnall, 1918	Romania	Ionescu 1951, 1955; Sendra et al. 2012
Campodea (Dicampa) apula Silvestri, 1912	Romania	Ionescu 1951, 1955
Campodea (Dicampa) campestris Ionescu, 1955	Bulgaria, Romania	Ionescu 1955; Sendra et al. 2012; Rusek 1965a
Campodea (Dicampa) caucasica Rusek, 1965	Bulgaria	Sendra and Georgiev 2021
Campodea (Dicampa) frenata Silvestri, 1912	Bulgaria, Romania	Ionescu 1951, 1955; Silvestri 1931; Paclt 1969
Campodea (Dicampa) malpighii Silvestri, 1912	Romania	Ionescu 1951, 1955
Campodea (Dicampa) propinqua Silvestri, 1932	Romania	Ionescu 1951, 1955
Campodea (Dicampa) silvicola Ionescu, 1955	Romania	Ionescu 1955
Campodea (Dicampa) sprovieri Silvestri, 1933	Bulgaria, Romania	Ionescu 1951, 1955; Sendra et al. 2012; Rusek 1965a; Sendra and Georgiev 2021
Campodea (Dicampa) transylvanica sp. nov.	Romania	This study
Campodea (Paurocampa) ruseki Rusek, 1965	Bulgaria	This study - new species for Bulgaria
Campodea (Paurocampa) suensoni Tuxen, 1930	Bulgaria & Romania	Ionescu 1951, 1955; Sendra et al. 2012; Rusek 1965b; Paclt 1969
*Eutrichocampacollina* Ionescu, 1955	Romania	Ionescu 1955; Sendra et al. 2012
*Litocampamontana* (Ionescu, 1955)	Romania	Ionescu 1955; Sendra et al. 2012
Plusiocampa (Plusiocampa) humicola Ionescu, 1951	Romania	Ionescu 1951, 1955; Sendra et al. 2012

The diversity of soil campodeids in Bulgaria and Romania is consistently higher than in regions at higher latitudes, which is expected due to the decrease in species numbers in northern areas (Sendra et al. 2021a, b). A clear example is the eleven *Campodea* s. str. species inhabiting the United Kingdom and Ireland, a region similar in area to Bulgaria and Romania together. Similarly, only four *Campodea* s. str. species are found in Norway and Sweden (Sendra and Reboleira 2020).

Another notable characteristic of the soil campodeid fauna in Romania and Bulgaria is its diversity in genera and subgenera, comprising four genera and three subgenera with a prevalence of species in the subgenus Dicampa. Almost 41% of all species belong to *Dicampa* (nine species). This richness is also high in the southern Mediterranean region, where *Dicampa* likely originated. For example, on the Iberian Peninsula, 30% of species belong to *Dicampa*, and in Morrocco, the figure is 36% (Sendra and Reboleira 2020).

The campodeid diversity in Romania (20 species) compared with Bulgaria (7 species) shows 25% overlap, with five species in common: C. (C.) magna, C. (D.) campestris, C. (D.) frenata, C. (D.) sprovieri, and C. (P.) suensoni. However, there is no overlap among the cave-adapted species, which typically have smaller ranges restricted to karstic areas in each region (Bareth and Condé 2001; Sendra et al. 2012).

Currently, three soil species can be considered as endemic: two are exclusive to Transylvania (Romania), *Plusiocampahumicola* and C. (D.) transylvanica Sendra, sp. nov., and one is found in Lotrului Mountains (Romania), *Litocampamontana*. All nine cave-adapted species are also endemic, with four limited to Romanian caves and five to Bulgarian caves.

Despite the extensive sampling efforts conducted in both countries, new taxonomic discoveries likely await entomologists and bioespeleologists who explore this primitive and fascinating group of Diplura.

## Supplementary Material

XML Treatment for Campodea (Campodea) magna

XML Treatment for Campodea (Campodea) plusiochaeta

XML Treatment for Campodea (Campodea) taunica

XML Treatment for Campodea (Campodea) wallacei

XML Treatment for Campodea (Dicampa) apula

XML Treatment for Campodea (Dicampa) campestris

XML Treatment for Campodea (Dicampa) frenata

XML Treatment for Campodea (Dicampa) propinqua

XML Treatment for Campodea (Dicampa) sprovierisprovieri

XML Treatment for Campodea (Dicampa) transylvanica

XML Treatment for Campodea (Paurocampa) ruseki

XML Treatment for Campodea (Paurocampa) suensoni

## ﻿Additional information

### Author ORCIDs

Alberto Sendra https://orcid.org/0000-0003-1035-3638

Cristina Fiera https://orcid.org/0000-0002-0125-5879

Jesús Selfa https://orcid.org/0000-0002-0376-7536

Pavel Stoev https://orcid.org/0000-0002-5702-5677

### Data availability

All of the data that support the findings of this study are available in the main text.

## References

[B1] Agrell I (1944) Die schwedischen Thysanuren. Opuscula entomologica 9: 23–36.

[B2] Arevad K (1957) Danske Diplura (Insecta, Apterygota). Entomologiske Meddelelser 28: 127–144.

[B3] Bagnall RS (1918) On two new species of *Campodea*. The Entomologist’s Monthly Magazine 54: 157–159.

[B4] Bareth C Condé B (1985) Campodéidés endogés de Ligurie (Diplura). Annali del Museo Civico di Storia Naturale “Giacomo Doria” 85: 251–258.

[B5] Bareth C Condé B (2001) Campodéidés des grottes de Bulgarie (Insecta: Diplura). Mémoires de Biospéologie 28: 9–27.

[B6] Blesić B (1984) Fauna Diplura (Insecta) Sr Srbije. Collection of Scientific Papers of the Faculty of Science Kragujevac 5: 91–96.

[B7] Blesić B (1997) Knowledge of Protura and Diplura of Montenegro. The Montenegrin Academy of Sciences and Arts Glasnik of the Section of Natural Sciences 12: 63–70.

[B8] Blesić B (1998a) Investigations of Protura and Diplura of South Serbia. Proceedings for Natural Sciences Matica Srpska 94: 87–90.

[B9] Blesić B (1998b) Knowledge of Protura and Diplura of Montenegro. The Montenegrin Academy of Sciences and Arts Glasnik of the Section of Natural Sciences 12: 63–70.

[B10] Blesić B. (2000a) Investigation of Diplura and Protura in western Serbia. Proceedings for Natural Sciences 99: 69–79. https://www.maticasrpska.org.rs/stariSajt/casopisi/prirodne_nauke_099.pdf

[B11] Blesić B (2000b) Of the Subgenus Paurocampa Silvestri, 1932 (Insecta: Diplura) on the Balkans and the rest of Europe. BIOS (Macedonia, Greece), Scientific Annals of the School of Biology 5: 23–26.

[B12] Blesić B (2001) Protura and Diplura (Insecta: Apterygota) of the Republic of Macedonia. 75 years Macedonian Museum Natural History: 157–162.

[B13] Christian E (1992) Verbreitung und Habitatpräferenz von Doppel- und Zangenschwänzen in der Großstadt Wien (Diplura: Campodeidae, Japygidae). Entomologia Generalis 17(3): 195–205. 10.1127/entom.gen/17/1992/195

[B14] Christian E (2003) Checklist of the Diplura of Germany. Entomofauna Germanica 6: 26–32.

[B15] Condé B (1947a) Campodéidés d’Algérie. Bulletin de la Société entomologique de France 52(9): 144–146. 10.3406/bsef.1947.15990

[B16] Condé B (1947b) Quelques campodéidés du nord-est de la France. Bulletin Société Sciences Nancy nouvelle série 6: 85–92.

[B17] Condé B (1947c) Nouvelles stations françaises de campodéidés avec description d’une forme nouvelle. Annales des Sciences naturelles Zoologie 11^ème^ série (9): 139–144.

[B18] Condé B (1950) Campodéidés du Var et des Alpes-Maritimes. Bulletin de la Société linnéenne de Lyon 6: 128–132. 10.3406/linly.1950.7321

[B19] Condé B (1953) Campodéidés endogés d’Afrique septentrionale. Bulletin de la Société zoologique de France 78(5–6): 358–377.

[B20] Condé B (1954) Ordnung: Entotropha (Diplura). In: Franz H (Eds) Die Nordost-Alpen im Spiegel ihrer Landtierwelt.Universitätsverlag Wagner, Innsbruck, 644–649.

[B21] Condé B (1956) Matériaux pour une monographie des Diploures Campodéidés. Mémoires du Muséum national d’histoire naturelle Série A – Zoologie 12: 1–202.

[B22] Condé B (1960) Protoures et Diploures Campodéidés des alluvions de la Moselle. Bulletin de la Société des Sciences de Nancy 19: 123–127.

[B23] Condé B (1961) Sur la microfaune du sol de Grande-Bretagne. II Diploures Campodédidés. Annales and Magazine of Natural History série 13, 4: 149–154. https://www.tandfonline.com/doi/pdf/10.1080/00222936108655794

[B24] Condé B (1962) Géonémie des Diploures troglobies du Jura et du Vercors. Spelunca Mémoires 2: 119–127.

[B25] Condé B (1966) Campodéidés de la région de Recoaro (Vénétie). Revue d’Écologie et de Biologie du sol 3(1): 166–169.

[B26] Condé B (1974) Les *Paurocampa* du groupe de *suensoni* Tuxen dans les grottes d’Europe centrale (Diploures Campodéidés). Revue suisse de Zoologie 81(2): 561–567. 10.5962/bhl.part.76023

[B27] Condé B (1984) Diploures Campodéidés (Insectes) de Grèce (1^ère^ note). Revue suisse de Zoologie 91(1): 173–201. 10.5962/bhl.part.81875

[B28] Condé B (1991) Campodéidés des Grottes de Bourgogne (Insectes, Diploures). Mémoires de Biospéologie 18: 243–246.

[B29] Condé B (1993) Une lignée danubienne du genre *Plusiocampa* (Diploures Campodéidés). Revue Suisse de Zoologie 100(3): 735–745. 10.5962/bhl.part.79881

[B30] Condé B (1996) Diploures Campodéidés de la Pestera de la Movile (Movile Cave), Dobroudja méridionales (Roumanie). Revue suisse de Zoologie 103(1): 101–114. 10.5962/bhl.part.79940

[B31] Drěnovski AK (1937) I prinos za izuěvane nissăta nasěkomna fauna – Apterygogenea na Bulgarija i Makadonija. Sofia, 6 pp.

[B32] Husson R (1946) Sur quelques récoltes de Diploures Campodéidés. Revue française d’Entomologie 13: 90–92.

[B33] Ionescu MA (1951) Contributiuni la studiul Campodeidelor din Republica Populară Română. Buletin Ştiinţific, Secțiunea de Știinţe, Agronomice, Geologice și Geografice 3(3): 525−532.

[B34] Ionescu MA (1955) Diplura. In: Fauna Republicii Populare Române, Insecta VII (3): 1−48.

[B35] Olsen KM (1996) Tohalen *Campodealubbockiii* Silvestri, 1912, “kranstohale”. Insekt-Nytt 21(4): 7–8.

[B36] Orelli M (1956) Untersuchungen zur postembryonalen Entwicklung von Campodea (Insecta, Apterygota). Verhandlungen der Naturforschenden Gesellschaft in Basel 67(3): 501–574.

[B37] Paclt J (1957) Diplura. Genera Insectorum 212E. P. Wytsman, Crainhem.

[B38] Paclt J (1961) Campodeidae des Senckenberg-Museums (Ins.-Diplura). Senckenbergiana biologica 42 (5/6): 455–458.

[B39] Paclt J (1965) Neue Beiträge zur Kenntnis der Apterygoten-Sammlung des Zoologischen Staatsinstituts und Zoologischen Museums Hamburg. Entomologische Mitteilungen aus dem Zoologischen Staatsinstitut und Zoologischen Museum Hamburg 3(54): 93–104.

[B40] Paclt J (1969) Über zwei verkannte *Campodea*-Arten (Insecta, Diplura) aus dem Witoscha bei Sofia. Zoologischer Anzeiger 182 (3/4): 285–287.

[B41] Paclt J Rusek J (1961) *Campodeasuensoni* Tuxen (Insecta, Diplura) clenem beskydské. Publications Faculty of Science University of Brno 415: 279–283.

[B42] Pagés J (1951) Contribution à la connaissance des Diploures. Supplément du Bulletin scientifique de Bourgogne 9: 1–97.

[B43] Racovitza EG (1907) Essai sur les problèmes biospéologiques. Biospeleologica, I. Archives de Zoologie experimentale et génerale premiere serie 6: 371–488.

[B44] Ramellini P (1990) Diplura dei Monti Ausoni e Aurunci (Lazio): Fauna ed Ecologia. Bollettino dell’Associazione Romana di Entomologia 44: 13–28.

[B45] Ramellini P (1995) Materiali per un catálogo topografico dei Dipluri Italiani. Fragmenta entomológica 27(1): 15–50.

[B46] Ramellini P (2000) Note su Campodeidi del Piemonte (Diplura, Campodeidae). Rivista Piemontese di Storia Naturale 21: 103–114.

[B47] Rusek J (1964) Über die Diplura (Apterygota) der Tschechoslowakei. Acta Societatis Zoologicae Bohemoslovenicae 28(2): 134–154.

[B48] Rusek J (1965a) Zur Kenntnis der Campodeidae (Diplura) Bulgariens. Acta entomologica Bohemoslovaca 62: 92–97.

[B49] Rusek J (1965b) Campodeids (Campodeidae, Diplura) of South-Eastern Europe. Zoologichesky Zhurnal 44(6): 1345–1356.

[B50] Sendra A (2023) Habitantes de la oscuridad: Fauna Ibero-balear de las cuevas. Sociedad Entomológica Aragonesa, 752 pp.

[B51] Sendra A Georgiev D (2021) Campodeinae (Campodeidae, Diplura) records from Sarnena Gora Mts, Bulgaria. In: Georgiev D, Bechev D, Yancheva V (Eds) Fauna of Sarnena Sredna Gora Mts, Part 2 ZooNotes, Supplement 10: 12−13.

[B52] Sendra A Moreno A (2004) El subgénero *Campodea**s.str.* en la Península Ibérica (Hexapoda: Diplura: Campodeidae). Boletin de la Sociedad Entomológica Aragonesa 35: 19–38. https://dialnet.unirioja.es/servlet/articulo?codigo=1047183

[B53] Sendra A Reboleira ASPS (2020) Euro-Mediterranean fauna of Campodeinae (Campodeidae, Diplura). European Journal of Taxonomy 728: 1–130. 10.5852/ejt.2020.728.1181

[B54] Sendra A Satar A Montagud S (2006) Première contribution à la faune de Diploures Campodéidés de la Péninsule d’Anatolie, Turquie (Diplura: Campodeidae). Revue suisse de Zoologie 113(3): 693–709. 10.5962/bhl.part.80368

[B55] Sendra A Teruel S Satar A Tusun S Özbay C (2010) New species, new records, and distribution of Campodeidae (Diplura) in Anatolia. Zootaxa 2639: 40–52. 10.11646/zootaxa.2639.1.4

[B56] Sendra A Nitzu E Sanjuan A (2012) Half a century after Ionescu’s work on Romanian Diplura – A faunal contribution based on material collected from karst areas. Travaux de l’Institut de Spéologie “Émile Racovitza” 51: 37–66. https://www.travaux-racovitza.com/journals/downloads/12/art02.pdf

[B57] Sendra A García Y Weber D (2013) Campodeidae (Hexapoda, Diplura) from caves of the Grand Duchy of Luxembourg. In: Weber D (Eds) Die Höhlenfauna Luxemburgs.Ferrantia 69, Musée national d’histoire naturelle, Luxembourg, 216–226. https://ps.mnhn.lu/ferrantia/publications/Ferrantia69/Ferrantia69%20216-226.pdf

[B58] Sendra A Jiménez-Valverde A Gilgado JD Ledesma E Baquero E Pérez-Suárez G Cuesta E Herrero-Borgoñón JJ Jordana R Tinaut A Barranco P Ortuño VM (2017) Diplurans of subsurface terrestrial habitats in the Iberian Peninsula, with a new species description (Diplura: Campodeidae). Zootaxa 4291(1): 61–80. 10.11646/zootaxa.4291.1.4

[B59] Sendra A Antić D Barranco P Borko Š Christian E Delić T Fadrique F Faille A Galli L Gasparo F Georgiev D Giachino PM Kováč L Lukić M Marcia P Miculinić K Nicolosi G Palero F Paragamian K Pérez T Polak S Prieto CE Turbanov I Vailati D Reboleira ASPS (2020) Flourishing in subterranean ecosystems: Euro-mediterranean Plusiocampinae and tachycampoids (Diplura, Campodeidae). European Journal of Taxonomy 591: 1–138. 10.5852/ejt.2020.728.1181

[B60] Sendra A Jiménez-Valverde A Selfa J Reboleira ASPS (2021a) Diversity, ecology, distribution and biogeography of Diplura. Insect Conservation and Diversity, 14: 415–425. 10.1111/icad.12480

[B61] Sendra A Palero F Jiménez-Valverde A Reboleira ASPS (2021b) Diplura in caves: diversity, ecology, evolution and biogeography. Zoological Journal of the Linnean Society 192: 675–689. 10.1093/zoolinnean/zlaa116

[B62] Silvestri F (1912) Contribuzione alla conoscenza dei Campodeidae (Thysanura) d’Europa. Bolletino del Laboratorio di Zoologia generale e agraria in Portici 6: 110–147.

[B63] Silvestri F (1931) Contributo alla conoscenza dei Campodeidae (Thysanura) delle grotte della Bulgaria. Bulletin des Institutions royales d’histoire naturelle à Sofia 4: 97–107.

[B64] Silvestri F (1932a) Campodeidae (Thysanura) de España (primera parte). Eos 8: 115–164.

[B65] Silvestri F (1932b) Descripción de cinco nuevas Campodea (Thys.) de Marruecos. Boletín de la Sociedad española de Historia Natural 32(1): 75–87.

[B66] Silvestri F (1933) Nuovi contributi alla conoscenza della fauna delle isole Italiane dell’Egeo. Bolletino del Laboratorio di Zoologia generale e agraria della R. Scuola superiore d’agricoltura in Portici 27: 61–111. https://publikationen.ub.uni-frankfurt.de/frontdoor/index/index/docId/15218

[B67] Stach J (1929) Verzeichnis der Apterygogenea Ungarns. Annales Historico-Naturales Musei Nationalis Hungarici, Budapest 26: 269–312. http://publication.nhmus.hu/annales/cikkreszletes.php?idhoz=468

[B68] Stach J (1964) Katalog Fauny Polski. XV. Apterygota. Polska Akademia Nauk, Instytut Zoologiczny, Warszawa.

[B69] Szeptycki A (1974) Diplura (Campodeidae) of the Ojców National Park in Poland. Bulletin entomologique de Pologne 46: 745–748.

[B70] Tuxen SL (1930) Einige Apterygoten aus Südeuropa nebst Beschreibung zwei neuer Arten von Thysanura. Entomologiske Meddelelser 17: 219–227.

[B71] Vellay I (1900) Ordo Apterygogenea. In: Paszlavszky J (Eds) : A Magyar Birodalom Állatvilága (Fauna Regni Hungariae) III.Arthropoda. Királyi Magyar Természettudományi Társulat, Budapest, 19–22.

[B72] Wygodzinsky PW (1941) Über eine neue *Campodea* und eine neue *Lepismachilis* aus Südeuropa. Entomologiske meddelelser 22: 137–141.

